# Tris­[4-(di­methyl­amino)­pyridine][tris(pyra­zol-1-yl)methane]­­ruthenium(II) bis­(hexa­fluorido­phosphate) diethyl ether monosolvate

**DOI:** 10.1107/S1600536813025245

**Published:** 2013-09-21

**Authors:** Benjamin J. Coe, James Raftery, Daniela Rusanova

**Affiliations:** a School of Chemistry, University of Manchester, Manchester M13 9PL, UK

## Abstract

In the title compound, [Ru(C_10_H_10_N_6_)(C_7_H_10_N_2_)_3_](PF_6_)_2_·C_4_H_10_O, the Ru^II^ cation is coordinated by one tris­(1-pyrazol­yl)methane (Tpm) and three dimethylaminopyridine (dmap) ligands in a slightly distorted octa­hedral geometry. The asymmetric unit consists of one complex cation, two hexa­fluorido­phosphate anions and one diethyl ether solvent mol­ecule in general positions. Although quite a large number of ruthenium complexes of the facially coordinating tridentate Tpm ligand have been structurally characterized, this is only the second one containing three pyridyl co-ligands. The average Ru—N(Tpm) distance is 2.059 (12) Å, while the average Ru—N(dmap) [dmap = 4-(di­methyl­amino)­pyridine] distance is somewhat longer at 2.108 (13) Å. The orientation of the dmap ligands varies greatly, with dihedral angles between the pyridyl and opposite pyrazolyl rings of 14.3 (2), 23.2 (2) and 61.2 (2)°.

## Related literature

For background to the synthesis, see: Llobet *et al.* (1988 ▶); Calvert *et al.* (1983 ▶). For examples of other structures of ruthenium complexes of the Tpm ligand, see: Llobet *et al.* (1989 ▶); Wilson & Nelson (2003 ▶); Katz *et al.* (2005 ▶); Iengo *et al.* (2005 ▶); Foxon *et al.* (2007 ▶); Kuzu *et al.* (2009 ▶); Waywell *et al.* (2010 ▶); Zagermann *et al.* (2011 ▶); De *et al.* (2011 ▶); Agarwala *et al.* (2011 ▶, 2013 ▶); Serrano *et al.* (2011 ▶); Cadranel *et al.* (2012 ▶). For examples of other structures of ruthenium complexes of the dmap ligand, see: Bonnet *et al.* (2003 ▶); Rossi *et al.* (2008 ▶, 2010 ▶); Mutoh *et al.* (2010 ▶); Dunbar *et al.* (2011 ▶). For the closest related structure, see: Laurent *et al.* (1999 ▶). 
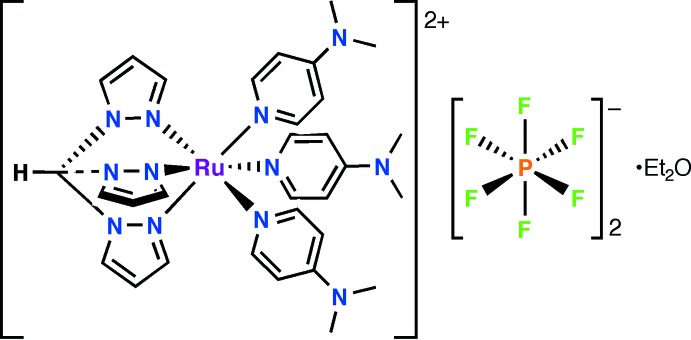


## Experimental

### 

#### Crystal data

[Ru(C_10_H_10_N_6_)(C_7_H_10_N_2_)_3_](PF_6_)_2_·C_4_H_10_O
*M* *_r_* = 1045.88
Triclinic, 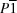

*a* = 12.1005 (9) Å
*b* = 12.5711 (9) Å
*c* = 15.7032 (11) Å
α = 80.047 (1)°
β = 75.377 (1)°
γ = 71.449 (1)°
*V* = 2180.1 (3) Å^3^
*Z* = 2
Mo *K*α radiation
μ = 0.53 mm^−1^
*T* = 100 K
0.30 × 0.10 × 0.03 mm

#### Data collection

Bruker SMART CCD area-detector diffractometer
19049 measured reflections
9932 independent reflections
7805 reflections with *I* > 2σ(*I*)
*R*_int_ = 0.051

#### Refinement

*R*[*F* ^2^ > 2σ(*F* ^2^)] = 0.057
*wR*(*F* ^2^) = 0.114
*S* = 0.95
9932 reflections
576 parameters
H-atom parameters constrained
Δρ_max_ = 1.03 e Å^−3^
Δρ_min_ = −0.98 e Å^−3^

### 

Data collection: *SMART* (Bruker, 2003 ▶); cell refinement: *SAINT-Plus* (Bruker, 2003 ▶); data reduction: *SAINT-Plus*; program(s) used to solve structure: *SHELXS97* (Sheldrick, 2008 ▶); program(s) used to refine structure: *SHELXL97* (Sheldrick, 2008 ▶); molecular graphics: *SHELXTL* (Sheldrick, 2008 ▶); software used to prepare material for publication: *SHELXTL*.

## Supplementary Material

Crystal structure: contains datablock(s) I. DOI: 10.1107/S1600536813025245/nc2317sup1.cif

Structure factors: contains datablock(s) I. DOI: 10.1107/S1600536813025245/nc2317Isup2.hkl

Additional supplementary materials:  crystallographic information; 3D view; checkCIF report

## Figures and Tables

**Table 1:** Selected bond lengths (Å)

N1—Ru1	2.122 (3)
N3—Ru1	2.097 (3)
N5—Ru1	2.104 (3)
N8—Ru1	2.071 (3)
N10—Ru1	2.048 (3)
N12—Ru1	2.059 (3)

**Fig. 1.**
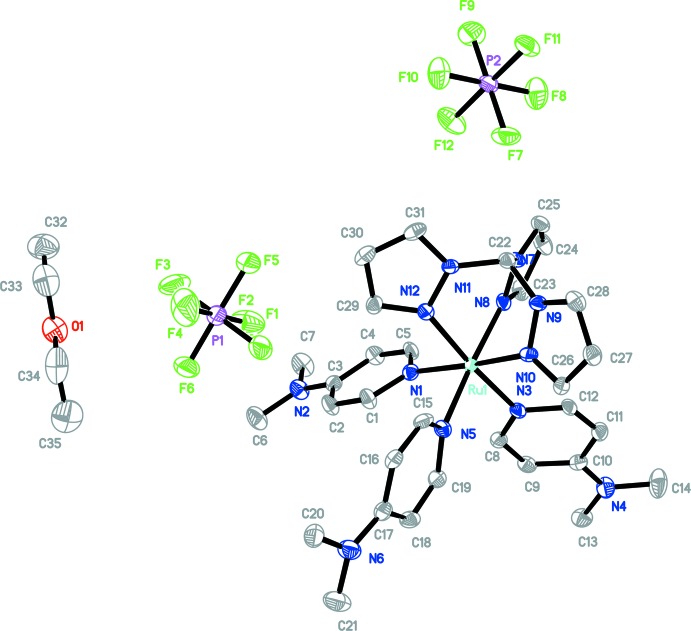
Crystal structure of the title compound with labeling and displacement ellipsoids drawn at the 50% probability level. Hydrogen atoms are omitted for clarity.

## supplementary crystallographic information

### 1. Comment

Ruthenium complexes of the tris(1-pyrazolyl)methane (Tpm) ligand have been studied in a number of laboratories, and many examples have been structurally characterized (*e.g.* Llobet *et al.*, 1989; Wilson & Nelson, 2003; Katz *et al.*, 2005; Iengo *et al.*, 2005; Foxon *et al.*, 2007; Kuzu *et al.*, 2009; Zagermann *et al.*, 2011; De *et al.*, 2011; Agarwala *et al.*, 2011; Serrano *et al.*, 2011; Cadranel *et al.*, 2012; Agarwala *et al.*, 2013). However, the only one featuring three pyridine (py) or pyridyl coligands is the complex salt [Ru^II^(Tpm)(py)_3_][PF_6_]_2_ (Laurent *et al.*, 1999), while [Ru^II^(Tpm)(dppz)(3-NH_2_py)][PF_6_]_2_·2MeCN·0.5H_2_O (dppz = dipyrido[3,2-*a*:2',3'-*c*]phenazine) (Waywell *et al.*, 2010) contains one chelating coligand.

The new compound (I) was synthesized simply by substituting all three chloride ligands in Ru^III^Cl_3_(Tpm) (Llobet *et al.*, 1988) with 4-(dimethylamino)pyridine (dmap) under reducing conditions, by adapting a method used previously to prepare [Ru^II^(Tpm)(vpy)_3_][PF_6_]_2_ (vpy = 4-vinylpyridine) (Calvert *et al.*, 1983). The isolated yield is reasonably high, while the blue colour is attributable to traces of the Ru(III) form of the complex which is rendered relatively electron-rich by the three dmap ligands. If a drop of ascorbic acid solution is added to an acetone solution of (I), the solution turns pale yellow immediately, indicating complete reduction to the Ru(II) species. The signals in the ^1^H NMR spectrum show no broadening, consistent with an adequately pure sample.

The complex salt (I) shows an intense, broad UV absorption band at λ_max_ = 322 nm in acetonitrile. This absorption is attributable to d→π* metal-to-ligand charge-transfer (MLCT) transitions from the Ru-based HOMO to the LUMOs localized on the dmap ligands. An additional band at λ_max_ = 264 nm is ascribed to ligand-based π→π* transitions, while a very weak band at λ_max_*ca* 590 nm is due to the blue-coloured Ru(III) form that disappears upon reduction with ascorbic acid. By way of comparison, the compound [Ru^II^(Tpm)(py)_3_][PF_6_]_2_ shows a MLCT band at 344 nm in acetonitrile; this is red-shifted when compared with that for (I) because the py ligands are more strongly electron-accepting than dmap.

Cyclic voltammetric studies on (I) reveal a reversible Ru^III/II^ wave at *E*_1/2_ = 0.75 V *versus*. Ag–AgCl, much lower than the value of 1.25 V for [Ru^II^(Tpm)(py)_3_][PF_6_]_2_ recorded under the same conditions (acetonitrile, 0.1 M [N(*n*-Bu_4_)]PF_6_, 100 mv s^-1^, ferrocene/ferrocenium standard at 0.44 V). This difference reflects the strong electron-donating ability of the dimethylamino substituents.

The molecular structure of the complex cation in (I) is as indicated by ^1^NMR spectroscopy, with a facially coordinating Tpm ligand and a slightly distorted octahedral coordination geometry. The N(Tpm)–Ru–N(Tpm) angles cover the range *ca* 85.3–86.5°, and the other angles at the Ru centre show small deviations from the ideal values. The average Ru–N(Tpm) distance of 2.059 (5) Å is similar to that reported for [Ru^II^(Tpm)(py)_3_][PF_6_]_2_ (2.074 (16) Å; Laurent *et al.*, 1999). The average Ru–N(dmap) distance of 2.108 (5) Å is the same as that reported for [Ru^II^(tpy)(phen)(dmap)][PF_6_]_2_ (tpy = 2,2';6',2''-terpyridine; phen = 1,10-phenanthroline) (2.107 (2) Å; Bonnet *et al.*, 2003), but a little shorter than that found in [Ru^II^(dmap)_6_]Cl_2_·6EtOH (2.131 (1) Å; Rossi *et al.*, 2008). A significantly shorter average Ru–N(dmap) distance has been reported for the trinuclear complex in *trans*-[(dmap)_4_Ru^II^{(µ-NC)Os^III^(CN)_5_}_2_][PPh_4_]_4_.10H_2_O (2.089 (13) Å; Rossi *et al.*, 2010). Considerably longer Ru–N(dmap) distances have been reported also, for example 2.333 (4) Å when positioned *trans* to a tellurocarbonyl ligand in *trans*,*cis*-Ru^II^Cl_2_(dmap)_2_(CTe)(H_2_IMes) (H_2_IMes = 1,3-dimesitylimidazolin-2-ylidene) (Mutoh *et al.*, 2010), and as long as 2.338 (3) Å when located *trans* to a carbene ligand in *trans*,*cis*-Ru^II^Cl_2_(dmap)_2_(PCy_3_){CH(C_6_H_4_)-4-NMe_2_} (Dunbar *et al.*, 2011).

The orientation of the dmap rings in (I) with respect to their opposite pyrazolyl rings is highly variable, with the following dihedral angles: 61.2° between N1/C1/C2/C3/C4/C5 and N9/N10/C26/C27/C28; 23.2° between N3/C8/C9/C10/C11/C12 and N11/N12/C29/C30/C31; 14.3° between N5/C15/C16/C17/C18/C19 and N7/N8/C23/C24/C25. A similar orientational variability of the py rings is found in [Ru^II^(Tpm)(py)_3_][PF_6_]_2_, with corresponding dihedral angles of 70.0, 20.6 and 10.2° (Laurent *et al.*, 1999).

### 2. Experimental

Ru^III^Cl_3_(Tpm)·1.5H_2_O (123 mg, 0.274 mmol), dmap (344 mg, 2.812 mmol) and 3:1 ethanol/water (degassed, 20 cm^3^) were heated at reflux under N_2_ for 18 h. As the temperature increased, the brown suspension became a blue-green colour. After cooling to room temperature, the solution was evaporated to a minimum volume and 0.1 M aqueous NH_4_PF_6_ (5 cm^3^) was added. The light-blue precipitate was filtered off, then dissolved through the glass sinter in acetone, removing a white residue. The acetone solution was evaporated to a minimum volume and diethyl ether was added, forming a blue oil. The diethyl ether was decanted off and the oil was dissolved in dichloromethane and washed (5 times) with water. The green dichloromethane layer was dried over MgSO_4_ and filtered through celite. The filtrate was evaporated to a minimum volume and diethyl ether was added. The pale blue precipitate was filtered off, washed with diethyl ether and dried. Yield: 199 mg (73%). Analysis calculated for C_31_H_40_F_12_N_12_P_2_Ru·0.3CH_2_Cl_2_: C 37.7, H 4.1, N 16.9%; found: C 37.5, H 3.7, N 16.6%. Spectroscopic analysis, ^1^H NMR (300 MHz, CD_3_COCD_3_, δ, p.p.m.) 9.79 (1*H*, s, CH), 8.70 (3*H*, d, J = 2.9 Hz, C_3_H_3_N_2_), 7.86 (6*H*, d, J = 7.1 Hz, C_5_H_4_N), 7.75 (3*H*, d, J = 1.7 Hz, C_3_H_3_N_2_), 6.74–6.63 (9*H*, C_3_H_3_N_2_ + C_5_H_4_N), 3.10 (18*H*, s, Me). ES–MS m/*z* = 827 ({*M* – PF_6_^-^}^+^), 681 ({*M* – 2PF_6_^-^}^+^), 341 ({*M* – 2PF_6_^-^}^2+^). Single crystals (pale yellow but coated in blue oil) suitable for X-ray diffraction studies were grown by slow diffusion of diethyl ether vapour into an acetone solution at room temperature.

### 3. Refinement

The structure was solved by direct methods. The H atoms were placed in calculated positions (methyl H atoms were allowed to rotate but not to tip) and were refined isotropically with *U*_iso_(H) = 1.2 *U*_eq_(C) (1.5 for methyl H atoms) using a riding model with C—H lengths of 0.95(CH), 0.99(CH_2_) & 0.98(CH_3_) Å.

### Crystal data

**Table:**

[Ru(C_10_H_10_N_6_)(C_7_H_10_N_2_)_3_](PF_6_)_2_·C_4_H_10_O	*Z* = 2
*M**_r_* = 1045.88	*F*(000) = 1068
Triclinic, *P*1	*D*_x_ = 1.593 Mg m^−^^3^
*a* = 12.1005 (9) Å	Mo *K*α radiation, λ = 0.71073 Å
*b* = 12.5711 (9) Å	Cell parameters from 3615 reflections
*c* = 15.7032 (11) Å	θ = 2.5–26.4°
α = 80.047 (1)°	µ = 0.53 mm^−^^1^
β = 75.377 (1)°	*T* = 100 K
γ = 71.449 (1)°	Plate, white
*V* = 2180.1 (3) Å^3^	0.30 × 0.10 × 0.03 mm

### Data collection

**Table:**

Bruker SMART CCD area-detector diffractometer	7805 reflections with *I* > 2σ(*I*)
Radiation source: fine-focus sealed tube	*R*_int_ = 0.051
Graphite monochromator	θ_max_ = 28.3°, θ_min_ = 1.7°
phi and ω scans	*h* = −15→15
19049 measured reflections	*k* = −16→16
9932 independent reflections	*l* = −20→20

### Refinement

**Table:**

Refinement on *F*^2^	Primary atom site location: structure-invariant direct methods
Least-squares matrix: full	Secondary atom site location: difference Fourier map
*R*[*F*^2^ > 2σ(*F*^2^)] = 0.057	Hydrogen site location: inferred from neighbouring sites
*wR*(*F*^2^) = 0.114	H-atom parameters constrained
*S* = 0.95	*w* = 1/[σ^2^(*F*_o_^2^) + (0.0406*P*)^2^] where *P* = (*F*_o_^2^ + 2*F*_c_^2^)/3
9932 reflections	(Δ/σ)_max_ = 0.001
576 parameters	Δρ_max_ = 1.03 e Å^−^^3^
0 restraints	Δρ_min_ = −0.98 e Å^−^^3^

### Special details

**Table:**

Geometry. All e.s.d.'s are estimated using the full covariance matrix. The cell e.s.d.'s are taken into account individually in the estimation of e.s.d.'s in distances, angles and torsion angles; correlations between e.s.d.'s in cell parameters are only used when they are defined by crystal symmetry
Refinement. Refinement of *F*^2^ against ALL reflections. The weighted *R*-factor *wR* and goodness of fit *S* are based on *F*^2^, conventional *R*-factors *R* are based on *F*, with *F* set to zero for negative *F*^2^. The threshold expression of *F*^2^ > σ(*F*^2^) is used only for calculating *R*-factors(gt) *etc*. and is not relevant to the choice of reflections for refinement. *R*-factors based on *F*^2^ are statistically about twice as large as those based on *F*, and *R*- factors based on ALL data will be even larger.

### Fractional atomic coordinates and isotropic or equivalent isotropic displacement parameters (Å^2^)

**Table:**

	*x*	*y*	*z*	*U*_iso_*/*U*_eq_	
C1	0.7382 (4)	0.1177 (3)	0.5113 (2)	0.0248 (9)	
H1	0.7583	0.1806	0.5220	0.030*	
C2	0.7017 (4)	0.0496 (3)	0.5832 (3)	0.0275 (9)	
H2	0.6981	0.0659	0.6409	0.033*	
C3	0.6696 (3)	−0.0442 (3)	0.5727 (3)	0.0234 (9)	
C4	0.6752 (4)	−0.0577 (3)	0.4850 (3)	0.0246 (9)	
H4	0.6522	−0.1180	0.4726	0.030*	
C5	0.7135 (3)	0.0148 (3)	0.4170 (2)	0.0229 (9)	
H5	0.7160	0.0018	0.3586	0.027*	
C6	0.6289 (4)	−0.0952 (4)	0.7321 (3)	0.0379 (11)	
H6A	0.7068	−0.0917	0.7370	0.057*	
H6B	0.6075	−0.1569	0.7734	0.057*	
H6C	0.5685	−0.0237	0.7462	0.057*	
C7	0.5928 (4)	−0.2053 (4)	0.6294 (3)	0.0377 (11)	
H7A	0.5194	−0.1734	0.6067	0.057*	
H7B	0.5767	−0.2509	0.6858	0.057*	
H7C	0.6538	−0.2529	0.5867	0.057*	
C8	1.0074 (3)	−0.0062 (3)	0.3353 (3)	0.0218 (8)	
H8	0.9646	−0.0109	0.3949	0.026*	
C9	1.1044 (3)	−0.0934 (3)	0.3096 (3)	0.0222 (8)	
H9	1.1266	−0.1567	0.3511	0.027*	
C10	1.1722 (3)	−0.0919 (3)	0.2237 (3)	0.0225 (8)	
C11	1.1297 (3)	0.0029 (3)	0.1662 (3)	0.0248 (9)	
H11	1.1695	0.0084	0.1059	0.030*	
C12	1.0311 (3)	0.0871 (3)	0.1973 (2)	0.0206 (8)	
H12	1.0052	0.1502	0.1567	0.025*	
C13	1.3138 (4)	−0.2704 (3)	0.2609 (3)	0.0307 (10)	
H13A	1.3159	−0.2419	0.3146	0.046*	
H13B	1.3941	−0.3154	0.2349	0.046*	
H13C	1.2594	−0.3175	0.2759	0.046*	
C14	1.3387 (4)	−0.1768 (4)	0.1074 (3)	0.0469 (13)	
H14A	1.2844	−0.1649	0.0675	0.070*	
H14B	1.3996	−0.2496	0.0998	0.070*	
H14C	1.3773	−0.1163	0.0936	0.070*	
C15	0.8290 (3)	0.3890 (3)	0.4188 (3)	0.0211 (8)	
H15	0.7741	0.4359	0.3844	0.025*	
C16	0.8523 (3)	0.4366 (3)	0.4819 (2)	0.0221 (8)	
H16	0.8135	0.5138	0.4902	0.027*	
C17	0.9334 (3)	0.3716 (3)	0.5346 (3)	0.0249 (9)	
C18	0.9956 (3)	0.2630 (3)	0.5096 (3)	0.0250 (9)	
H18	1.0590	0.2176	0.5370	0.030*	
C19	0.9656 (3)	0.2218 (3)	0.4458 (2)	0.0216 (8)	
H19	1.0091	0.1473	0.4317	0.026*	
C20	0.8716 (4)	0.5209 (4)	0.6324 (3)	0.0329 (10)	
H20A	0.8780	0.5811	0.5843	0.049*	
H20B	0.8947	0.5366	0.6834	0.049*	
H20C	0.7892	0.5170	0.6495	0.049*	
C21	1.0310 (4)	0.3439 (4)	0.6590 (3)	0.0396 (12)	
H21A	0.9926	0.2918	0.7002	0.059*	
H21B	1.0501	0.3923	0.6924	0.059*	
H21C	1.1046	0.3009	0.6222	0.059*	
C22	0.6585 (3)	0.3694 (3)	0.1915 (2)	0.0188 (8)	
H22	0.6096	0.4206	0.1499	0.023*	
C23	0.7489 (4)	0.0752 (3)	0.1931 (2)	0.0250 (9)	
H23	0.7897	0.0010	0.2138	0.030*	
C24	0.6901 (4)	0.1006 (3)	0.1235 (3)	0.0291 (10)	
H24	0.6831	0.0489	0.0890	0.035*	
C25	0.6447 (3)	0.2147 (3)	0.1149 (3)	0.0236 (9)	
H25	0.5996	0.2584	0.0728	0.028*	
C26	0.9455 (3)	0.3760 (3)	0.1926 (2)	0.0205 (8)	
H26	1.0177	0.3498	0.2135	0.025*	
C27	0.9210 (4)	0.4656 (3)	0.1276 (3)	0.0239 (9)	
H27	0.9714	0.5106	0.0969	0.029*	
C28	0.8095 (4)	0.4752 (3)	0.1172 (2)	0.0230 (9)	
H28	0.7666	0.5292	0.0779	0.028*	
C29	0.5636 (3)	0.3661 (3)	0.4219 (3)	0.0208 (8)	
H29	0.5735	0.3399	0.4808	0.025*	
C30	0.4624 (3)	0.4454 (3)	0.3999 (3)	0.0259 (9)	
H30	0.3925	0.4827	0.4394	0.031*	
C31	0.4845 (3)	0.4584 (3)	0.3095 (3)	0.0248 (9)	
H31	0.4322	0.5065	0.2736	0.030*	
C32	−0.0178 (5)	0.1634 (5)	0.9442 (4)	0.0647 (17)	
H32A	0.0243	0.1048	0.9040	0.097*	
H32B	−0.0881	0.2136	0.9229	0.097*	
H32C	−0.0430	0.1281	1.0036	0.097*	
C33	0.0611 (5)	0.2281 (4)	0.9471 (4)	0.0560 (15)	
H33A	0.0845	0.2658	0.8874	0.067*	
H33B	0.0185	0.2873	0.9877	0.067*	
C34	0.2419 (5)	0.2204 (4)	0.9812 (4)	0.0560 (16)	
H34A	0.1999	0.2774	1.0238	0.067*	
H34B	0.2650	0.2604	0.9225	0.067*	
C35	0.3515 (5)	0.1424 (5)	1.0105 (4)	0.0682 (18)	
H35A	0.3281	0.0998	1.0670	0.102*	
H35B	0.4016	0.1866	1.0178	0.102*	
H35C	0.3963	0.0900	0.9658	0.102*	
F1	0.4903 (2)	0.2958 (2)	0.63159 (16)	0.0468 (7)	
F2	0.4247 (3)	0.1426 (2)	0.6560 (2)	0.0637 (9)	
F3	0.2520 (2)	0.2383 (3)	0.7375 (2)	0.0811 (12)	
F4	0.3175 (3)	0.3913 (3)	0.7116 (2)	0.0787 (11)	
F5	0.3163 (2)	0.3063 (2)	0.59690 (17)	0.0450 (7)	
F6	0.4260 (2)	0.2253 (3)	0.77040 (17)	0.0532 (8)	
F7	0.4333 (2)	0.5084 (2)	0.11112 (17)	0.0446 (7)	
F8	0.3946 (2)	0.3930 (2)	0.03370 (17)	0.0482 (7)	
F9	0.1992 (2)	0.4556 (2)	0.09274 (17)	0.0458 (7)	
F10	0.2368 (2)	0.5714 (2)	0.16910 (17)	0.0455 (7)	
F11	0.3025 (2)	0.5791 (2)	0.02154 (15)	0.0354 (6)	
F12	0.3306 (3)	0.3853 (2)	0.18237 (17)	0.0495 (8)	
N1	0.7480 (3)	0.1031 (3)	0.4263 (2)	0.0194 (7)	
N2	0.6349 (3)	−0.1148 (3)	0.6426 (2)	0.0284 (8)	
N3	0.9678 (3)	0.0875 (3)	0.2810 (2)	0.0195 (7)	
N4	1.2724 (3)	−0.1759 (3)	0.1975 (2)	0.0296 (8)	
N5	0.8787 (3)	0.2796 (3)	0.4016 (2)	0.0203 (7)	
N6	0.9507 (3)	0.4134 (3)	0.6027 (2)	0.0300 (8)	
N7	0.6755 (3)	0.2541 (2)	0.1773 (2)	0.0189 (7)	
N8	0.7408 (3)	0.1685 (3)	0.2272 (2)	0.0194 (7)	
N9	0.7719 (3)	0.3937 (3)	0.1732 (2)	0.0180 (7)	
N10	0.8545 (3)	0.3320 (3)	0.2215 (2)	0.0197 (7)	
N11	0.5944 (3)	0.3900 (2)	0.2808 (2)	0.0176 (7)	
N12	0.6450 (3)	0.3315 (3)	0.3498 (2)	0.0189 (7)	
O1	0.1650 (3)	0.1576 (3)	0.9763 (2)	0.0442 (8)	
P1	0.37068 (10)	0.26663 (11)	0.68477 (8)	0.0332 (3)	
P2	0.31577 (10)	0.48166 (9)	0.10162 (7)	0.0247 (2)	
Ru1	0.80863 (3)	0.21377 (3)	0.31963 (2)	0.01498 (8)	

### Atomic displacement parameters (Å^2^)

**Table:**

	*U*^11^	*U*^22^	*U*^33^	*U*^12^	*U*^13^	*U*^23^
C1	0.034 (2)	0.028 (2)	0.018 (2)	−0.0160 (19)	−0.0042 (17)	−0.0023 (17)
C2	0.037 (3)	0.034 (2)	0.016 (2)	−0.019 (2)	−0.0055 (18)	0.0012 (18)
C3	0.019 (2)	0.027 (2)	0.022 (2)	−0.0071 (17)	−0.0044 (16)	0.0026 (17)
C4	0.031 (2)	0.020 (2)	0.026 (2)	−0.0109 (18)	−0.0069 (18)	−0.0029 (17)
C5	0.027 (2)	0.026 (2)	0.0153 (19)	−0.0056 (18)	−0.0050 (16)	−0.0056 (16)
C6	0.054 (3)	0.043 (3)	0.024 (2)	−0.029 (2)	−0.009 (2)	0.008 (2)
C7	0.056 (3)	0.030 (2)	0.035 (3)	−0.025 (2)	−0.011 (2)	0.003 (2)
C8	0.021 (2)	0.022 (2)	0.021 (2)	−0.0054 (16)	−0.0062 (16)	0.0020 (16)
C9	0.025 (2)	0.0164 (19)	0.023 (2)	−0.0013 (16)	−0.0100 (17)	0.0016 (16)
C10	0.020 (2)	0.022 (2)	0.027 (2)	−0.0040 (16)	−0.0076 (17)	−0.0049 (17)
C11	0.022 (2)	0.025 (2)	0.021 (2)	−0.0012 (17)	−0.0025 (17)	−0.0008 (17)
C12	0.021 (2)	0.021 (2)	0.0153 (19)	−0.0019 (16)	−0.0065 (16)	0.0029 (16)
C13	0.028 (2)	0.024 (2)	0.037 (3)	0.0013 (18)	−0.0105 (19)	−0.0026 (19)
C14	0.043 (3)	0.039 (3)	0.036 (3)	0.006 (2)	0.009 (2)	−0.004 (2)
C15	0.0135 (18)	0.025 (2)	0.026 (2)	−0.0067 (16)	−0.0069 (16)	0.0018 (17)
C16	0.0162 (19)	0.025 (2)	0.024 (2)	−0.0071 (16)	0.0005 (16)	−0.0036 (17)
C17	0.021 (2)	0.034 (2)	0.021 (2)	−0.0157 (18)	−0.0018 (17)	0.0021 (18)
C18	0.021 (2)	0.027 (2)	0.026 (2)	−0.0066 (17)	−0.0096 (17)	0.0074 (18)
C19	0.021 (2)	0.021 (2)	0.021 (2)	−0.0051 (16)	−0.0050 (16)	0.0050 (16)
C20	0.028 (2)	0.046 (3)	0.027 (2)	−0.015 (2)	0.0007 (19)	−0.013 (2)
C21	0.044 (3)	0.051 (3)	0.035 (3)	−0.026 (2)	−0.020 (2)	0.007 (2)
C22	0.0185 (19)	0.0182 (19)	0.0179 (19)	−0.0011 (15)	−0.0056 (15)	−0.0023 (15)
C23	0.031 (2)	0.019 (2)	0.020 (2)	−0.0052 (17)	−0.0036 (17)	0.0017 (16)
C24	0.046 (3)	0.024 (2)	0.021 (2)	−0.015 (2)	−0.0078 (19)	−0.0037 (17)
C25	0.026 (2)	0.028 (2)	0.020 (2)	−0.0103 (18)	−0.0089 (17)	−0.0015 (17)
C26	0.0173 (19)	0.024 (2)	0.0175 (19)	−0.0034 (16)	−0.0022 (15)	−0.0016 (16)
C27	0.028 (2)	0.024 (2)	0.020 (2)	−0.0105 (18)	−0.0037 (17)	0.0002 (17)
C28	0.031 (2)	0.0153 (19)	0.021 (2)	−0.0039 (17)	−0.0084 (17)	0.0029 (16)
C29	0.019 (2)	0.025 (2)	0.020 (2)	−0.0107 (17)	−0.0009 (16)	−0.0037 (16)
C30	0.016 (2)	0.027 (2)	0.035 (2)	−0.0058 (17)	−0.0015 (17)	−0.0104 (19)
C31	0.0157 (19)	0.019 (2)	0.037 (2)	0.0006 (16)	−0.0072 (17)	−0.0050 (18)
C32	0.064 (4)	0.051 (4)	0.078 (4)	−0.001 (3)	−0.031 (3)	−0.008 (3)
C33	0.082 (4)	0.030 (3)	0.047 (3)	−0.010 (3)	−0.010 (3)	0.003 (2)
C34	0.080 (4)	0.037 (3)	0.050 (3)	−0.033 (3)	0.015 (3)	−0.014 (3)
C35	0.069 (4)	0.065 (4)	0.085 (5)	−0.043 (4)	−0.005 (4)	−0.016 (4)
F1	0.0441 (17)	0.072 (2)	0.0310 (15)	−0.0305 (15)	−0.0070 (13)	0.0029 (14)
F2	0.069 (2)	0.0452 (19)	0.084 (2)	−0.0043 (16)	−0.0411 (19)	−0.0128 (17)
F3	0.0309 (17)	0.146 (3)	0.055 (2)	−0.033 (2)	−0.0158 (15)	0.044 (2)
F4	0.092 (3)	0.060 (2)	0.063 (2)	0.0124 (19)	−0.010 (2)	−0.0282 (18)
F5	0.0372 (16)	0.0582 (18)	0.0358 (15)	−0.0088 (14)	−0.0133 (13)	0.0038 (14)
F6	0.0439 (17)	0.087 (2)	0.0326 (16)	−0.0264 (16)	−0.0167 (13)	0.0107 (15)
F7	0.0339 (15)	0.0624 (19)	0.0471 (17)	−0.0187 (14)	−0.0236 (13)	0.0003 (14)
F8	0.0586 (19)	0.0390 (16)	0.0414 (17)	−0.0108 (14)	0.0025 (14)	−0.0148 (13)
F9	0.0405 (16)	0.0616 (19)	0.0482 (17)	−0.0324 (14)	−0.0149 (13)	0.0022 (14)
F10	0.0530 (18)	0.0419 (16)	0.0365 (16)	−0.0129 (14)	0.0033 (13)	−0.0113 (13)
F11	0.0413 (15)	0.0402 (15)	0.0302 (14)	−0.0187 (12)	−0.0179 (12)	0.0105 (12)
F12	0.071 (2)	0.0390 (16)	0.0378 (16)	−0.0171 (15)	−0.0236 (15)	0.0167 (13)
N1	0.0181 (16)	0.0179 (16)	0.0202 (17)	−0.0031 (13)	−0.0032 (13)	−0.0024 (13)
N2	0.038 (2)	0.0274 (19)	0.0232 (19)	−0.0166 (16)	−0.0074 (16)	0.0036 (15)
N3	0.0191 (17)	0.0204 (17)	0.0162 (16)	−0.0011 (13)	−0.0062 (13)	0.0002 (13)
N4	0.029 (2)	0.0246 (19)	0.0260 (19)	0.0021 (15)	−0.0030 (15)	−0.0007 (15)
N5	0.0219 (17)	0.0219 (17)	0.0161 (16)	−0.0080 (14)	−0.0054 (13)	0.0058 (13)
N6	0.030 (2)	0.037 (2)	0.028 (2)	−0.0152 (17)	−0.0107 (16)	−0.0010 (17)
N7	0.0199 (17)	0.0173 (16)	0.0184 (17)	−0.0016 (13)	−0.0083 (13)	0.0005 (13)
N8	0.0199 (17)	0.0186 (16)	0.0148 (16)	−0.0018 (13)	−0.0024 (13)	0.0016 (13)
N9	0.0180 (16)	0.0180 (16)	0.0180 (16)	−0.0046 (13)	−0.0066 (13)	0.0011 (13)
N10	0.0162 (16)	0.0203 (17)	0.0206 (17)	−0.0014 (13)	−0.0079 (13)	0.0016 (13)
N11	0.0149 (16)	0.0192 (16)	0.0183 (16)	−0.0028 (13)	−0.0058 (13)	−0.0018 (13)
N12	0.0191 (17)	0.0201 (17)	0.0174 (16)	−0.0048 (13)	−0.0044 (13)	−0.0023 (13)
O1	0.059 (2)	0.0349 (19)	0.040 (2)	−0.0188 (17)	−0.0111 (17)	0.0052 (15)
P1	0.0269 (6)	0.0413 (7)	0.0265 (6)	−0.0052 (5)	−0.0055 (5)	0.0007 (5)
P2	0.0261 (6)	0.0282 (6)	0.0216 (6)	−0.0085 (5)	−0.0097 (5)	0.0010 (5)
Ru1	0.01359 (15)	0.01514 (15)	0.01397 (15)	−0.00199 (11)	−0.00331 (11)	0.00089 (11)

### Geometric parameters (Å, º)

**Table:**

C1—N1	1.348 (5)	C22—N9	1.447 (4)
C1—C2	1.362 (5)	C22—H22	1.0000
C1—H1	0.9500	C23—N8	1.338 (5)
C2—C3	1.400 (5)	C23—C24	1.389 (5)
C2—H2	0.9500	C23—H23	0.9500
C3—N2	1.354 (5)	C24—C25	1.358 (5)
C3—C4	1.399 (5)	C24—H24	0.9500
C4—C5	1.367 (5)	C25—N7	1.348 (4)
C4—H4	0.9500	C25—H25	0.9500
C5—N1	1.346 (5)	C26—N10	1.333 (4)
C5—H5	0.9500	C26—C27	1.393 (5)
C6—N2	1.448 (5)	C26—H26	0.9500
C6—H6A	0.9800	C27—C28	1.365 (5)
C6—H6B	0.9800	C27—H27	0.9500
C6—H6C	0.9800	C28—N9	1.347 (4)
C7—N2	1.450 (5)	C28—H28	0.9500
C7—H7A	0.9800	C29—N12	1.333 (4)
C7—H7B	0.9800	C29—C30	1.389 (5)
C7—H7C	0.9800	C29—H29	0.9500
C8—C9	1.355 (5)	C30—C31	1.368 (5)
C8—N3	1.358 (4)	C30—H30	0.9500
C8—H8	0.9500	C31—N11	1.347 (4)
C9—C10	1.391 (5)	C31—H31	0.9500
C9—H9	0.9500	C32—C33	1.451 (7)
C10—N4	1.356 (5)	C32—H32A	0.9800
C10—C11	1.405 (5)	C32—H32B	0.9800
C11—C12	1.365 (5)	C32—H32C	0.9800
C11—H11	0.9500	C33—O1	1.421 (6)
C12—N3	1.343 (4)	C33—H33A	0.9900
C12—H12	0.9500	C33—H33B	0.9900
C13—N4	1.455 (5)	C34—O1	1.421 (6)
C13—H13A	0.9800	C34—C35	1.501 (7)
C13—H13B	0.9800	C34—H34A	0.9900
C13—H13C	0.9800	C34—H34B	0.9900
C14—N4	1.440 (5)	C35—H35A	0.9800
C14—H14A	0.9800	C35—H35B	0.9800
C14—H14B	0.9800	C35—H35C	0.9800
C14—H14C	0.9800	F1—P1	1.597 (3)
C15—N5	1.358 (5)	F2—P1	1.584 (3)
C15—C16	1.369 (5)	F3—P1	1.580 (3)
C15—H15	0.9500	F4—P1	1.580 (3)
C16—C17	1.408 (5)	F5—P1	1.612 (3)
C16—H16	0.9500	F6—P1	1.587 (3)
C17—N6	1.357 (5)	F7—P2	1.608 (3)
C17—C18	1.403 (5)	F8—P2	1.584 (3)
C18—C19	1.374 (5)	F9—P2	1.590 (3)
C18—H18	0.9500	F10—P2	1.589 (3)
C19—N5	1.350 (4)	F11—P2	1.598 (2)
C19—H19	0.9500	F12—P2	1.598 (3)
C20—N6	1.459 (5)	N1—Ru1	2.122 (3)
C20—H20A	0.9800	N3—Ru1	2.097 (3)
C20—H20B	0.9800	N5—Ru1	2.104 (3)
C20—H20C	0.9800	N7—N8	1.370 (4)
C21—N6	1.454 (5)	N8—Ru1	2.071 (3)
C21—H21A	0.9800	N9—N10	1.364 (4)
C21—H21B	0.9800	N10—Ru1	2.048 (3)
C21—H21C	0.9800	N11—N12	1.365 (4)
C22—N11	1.443 (4)	N12—Ru1	2.059 (3)
C22—N7	1.446 (5)		
			
N1—C1—C2	125.4 (4)	C29—C30—H30	127.2
N1—C1—H1	117.3	N11—C31—C30	107.1 (3)
C2—C1—H1	117.3	N11—C31—H31	126.5
C1—C2—C3	120.5 (4)	C30—C31—H31	126.5
C1—C2—H2	119.7	C33—C32—H32A	109.5
C3—C2—H2	119.7	C33—C32—H32B	109.5
N2—C3—C2	122.1 (4)	H32A—C32—H32B	109.5
N2—C3—C4	123.4 (4)	C33—C32—H32C	109.5
C2—C3—C4	114.5 (4)	H32A—C32—H32C	109.5
C5—C4—C3	120.7 (4)	H32B—C32—H32C	109.5
C5—C4—H4	119.6	O1—C33—C32	111.0 (4)
C3—C4—H4	119.6	O1—C33—H33A	109.4
N1—C5—C4	125.0 (4)	C32—C33—H33A	109.4
N1—C5—H5	117.5	O1—C33—H33B	109.4
C4—C5—H5	117.5	C32—C33—H33B	109.4
N2—C6—H6A	109.5	H33A—C33—H33B	108.0
N2—C6—H6B	109.5	O1—C34—C35	109.8 (4)
H6A—C6—H6B	109.5	O1—C34—H34A	109.7
N2—C6—H6C	109.5	C35—C34—H34A	109.7
H6A—C6—H6C	109.5	O1—C34—H34B	109.7
H6B—C6—H6C	109.5	C35—C34—H34B	109.7
N2—C7—H7A	109.5	H34A—C34—H34B	108.2
N2—C7—H7B	109.5	C34—C35—H35A	109.5
H7A—C7—H7B	109.5	C34—C35—H35B	109.5
N2—C7—H7C	109.5	H35A—C35—H35B	109.5
H7A—C7—H7C	109.5	C34—C35—H35C	109.5
H7B—C7—H7C	109.5	H35A—C35—H35C	109.5
C9—C8—N3	123.9 (4)	H35B—C35—H35C	109.5
C9—C8—H8	118.0	C5—N1—C1	113.7 (3)
N3—C8—H8	118.0	C5—N1—Ru1	124.4 (3)
C8—C9—C10	121.3 (4)	C1—N1—Ru1	121.9 (2)
C8—C9—H9	119.4	C3—N2—C7	120.2 (3)
C10—C9—H9	119.4	C3—N2—C6	120.8 (3)
N4—C10—C9	122.3 (4)	C7—N2—C6	118.8 (3)
N4—C10—C11	122.5 (4)	C12—N3—C8	114.7 (3)
C9—C10—C11	115.3 (3)	C12—N3—Ru1	122.1 (2)
C12—C11—C10	119.8 (4)	C8—N3—Ru1	122.8 (2)
C12—C11—H11	120.1	C10—N4—C14	122.0 (3)
C10—C11—H11	120.1	C10—N4—C13	119.9 (3)
N3—C12—C11	125.0 (3)	C14—N4—C13	118.1 (3)
N3—C12—H12	117.5	C19—N5—C15	114.1 (3)
C11—C12—H12	117.5	C19—N5—Ru1	126.5 (3)
N4—C13—H13A	109.5	C15—N5—Ru1	119.2 (2)
N4—C13—H13B	109.5	C17—N6—C21	121.2 (4)
H13A—C13—H13B	109.5	C17—N6—C20	120.2 (3)
N4—C13—H13C	109.5	C21—N6—C20	117.4 (4)
H13A—C13—H13C	109.5	C25—N7—N8	111.6 (3)
H13B—C13—H13C	109.5	C25—N7—C22	129.3 (3)
N4—C14—H14A	109.5	N8—N7—C22	118.8 (3)
N4—C14—H14B	109.5	C23—N8—N7	104.1 (3)
H14A—C14—H14B	109.5	C23—N8—Ru1	138.5 (3)
N4—C14—H14C	109.5	N7—N8—Ru1	117.2 (2)
H14A—C14—H14C	109.5	C28—N9—N10	111.6 (3)
H14B—C14—H14C	109.5	C28—N9—C22	129.7 (3)
N5—C15—C16	124.8 (4)	N10—N9—C22	118.5 (3)
N5—C15—H15	117.6	C26—N10—N9	104.7 (3)
C16—C15—H15	117.6	C26—N10—Ru1	137.0 (3)
C15—C16—C17	120.3 (4)	N9—N10—Ru1	118.0 (2)
C15—C16—H16	119.9	C31—N11—N12	111.4 (3)
C17—C16—H16	119.9	C31—N11—C22	129.4 (3)
N6—C17—C18	123.5 (4)	N12—N11—C22	119.2 (3)
N6—C17—C16	121.7 (4)	C29—N12—N11	104.7 (3)
C18—C17—C16	114.8 (4)	C29—N12—Ru1	137.9 (3)
C19—C18—C17	120.5 (4)	N11—N12—Ru1	117.2 (2)
C19—C18—H18	119.8	C33—O1—C34	111.6 (4)
C17—C18—H18	119.8	F4—P1—F3	90.4 (2)
N5—C19—C18	124.7 (4)	F4—P1—F2	178.9 (2)
N5—C19—H19	117.7	F3—P1—F2	90.3 (2)
C18—C19—H19	117.7	F4—P1—F6	91.35 (18)
N6—C20—H20A	109.5	F3—P1—F6	90.14 (15)
N6—C20—H20B	109.5	F2—P1—F6	89.47 (17)
H20A—C20—H20B	109.5	F4—P1—F1	89.37 (19)
N6—C20—H20C	109.5	F3—P1—F1	179.8 (2)
H20A—C20—H20C	109.5	F2—P1—F1	89.91 (17)
H20B—C20—H20C	109.5	F6—P1—F1	89.88 (14)
N6—C21—H21A	109.5	F4—P1—F5	89.74 (17)
N6—C21—H21B	109.5	F3—P1—F5	90.22 (15)
H21A—C21—H21B	109.5	F2—P1—F5	89.44 (16)
N6—C21—H21C	109.5	F6—P1—F5	178.85 (18)
H21A—C21—H21C	109.5	F1—P1—F5	89.76 (14)
H21B—C21—H21C	109.5	F8—P2—F10	179.54 (16)
N11—C22—N7	110.1 (3)	F8—P2—F9	89.91 (16)
N11—C22—N9	110.9 (3)	F10—P2—F9	90.15 (15)
N7—C22—N9	110.7 (3)	F8—P2—F12	90.61 (15)
N11—C22—H22	108.3	F10—P2—F12	89.85 (15)
N7—C22—H22	108.3	F9—P2—F12	90.19 (15)
N9—C22—H22	108.3	F8—P2—F11	89.81 (14)
N8—C23—C24	111.3 (4)	F10—P2—F11	89.73 (14)
N8—C23—H23	124.3	F9—P2—F11	90.88 (13)
C24—C23—H23	124.3	F12—P2—F11	178.86 (15)
C25—C24—C23	105.8 (4)	F8—P2—F7	90.42 (16)
C25—C24—H24	127.1	F10—P2—F7	89.52 (15)
C23—C24—H24	127.1	F9—P2—F7	179.66 (18)
N7—C25—C24	107.2 (3)	F12—P2—F7	89.72 (15)
N7—C25—H25	126.4	F11—P2—F7	89.21 (14)
C24—C25—H25	126.4	N10—Ru1—N12	86.48 (12)
N10—C26—C27	111.0 (3)	N10—Ru1—N8	85.34 (12)
N10—C26—H26	124.5	N12—Ru1—N8	86.28 (12)
C27—C26—H26	124.5	N10—Ru1—N3	93.90 (12)
C28—C27—C26	105.8 (3)	N12—Ru1—N3	174.30 (12)
C28—C27—H27	127.1	N8—Ru1—N3	88.08 (12)
C26—C27—H27	127.1	N10—Ru1—N5	87.03 (12)
N9—C28—C27	106.9 (3)	N12—Ru1—N5	91.45 (12)
N9—C28—H28	126.5	N8—Ru1—N5	172.16 (12)
C27—C28—H28	126.5	N3—Ru1—N5	94.25 (12)
N12—C29—C30	111.3 (4)	N10—Ru1—N1	174.85 (12)
N12—C29—H29	124.4	N12—Ru1—N1	88.56 (12)
C30—C29—H29	124.4	N8—Ru1—N1	95.73 (12)
C31—C30—C29	105.5 (3)	N3—Ru1—N1	91.17 (11)
C31—C30—H30	127.2	N5—Ru1—N1	91.71 (12)
			
N1—C1—C2—C3	−0.5 (7)	C30—C31—N11—C22	177.8 (3)
C1—C2—C3—N2	178.9 (4)	N7—C22—N11—C31	−113.9 (4)
C1—C2—C3—C4	−1.8 (6)	N9—C22—N11—C31	123.2 (4)
N2—C3—C4—C5	−178.5 (4)	N7—C22—N11—N12	63.2 (4)
C2—C3—C4—C5	2.1 (6)	N9—C22—N11—N12	−59.8 (4)
C3—C4—C5—N1	−0.2 (6)	C30—C29—N12—N11	0.0 (4)
N3—C8—C9—C10	0.6 (6)	C30—C29—N12—Ru1	−174.8 (3)
C8—C9—C10—N4	177.2 (4)	C31—N11—N12—C29	−0.4 (4)
C8—C9—C10—C11	−2.6 (6)	C22—N11—N12—C29	−177.9 (3)
N4—C10—C11—C12	−177.2 (4)	C31—N11—N12—Ru1	175.7 (2)
C9—C10—C11—C12	2.5 (6)	C22—N11—N12—Ru1	−1.8 (4)
C10—C11—C12—N3	−0.5 (6)	C32—C33—O1—C34	−178.8 (5)
N5—C15—C16—C17	0.3 (6)	C35—C34—O1—C33	−178.8 (4)
C15—C16—C17—N6	−174.2 (3)	C26—N10—Ru1—N12	131.7 (4)
C15—C16—C17—C18	7.3 (5)	N9—N10—Ru1—N12	−41.4 (3)
N6—C17—C18—C19	173.5 (4)	C26—N10—Ru1—N8	−141.7 (4)
C16—C17—C18—C19	−8.0 (5)	N9—N10—Ru1—N8	45.1 (3)
C17—C18—C19—N5	1.2 (6)	C26—N10—Ru1—N3	−54.0 (4)
N8—C23—C24—C25	0.2 (5)	N9—N10—Ru1—N3	132.9 (3)
C23—C24—C25—N7	−0.2 (5)	C26—N10—Ru1—N5	40.1 (4)
N10—C26—C27—C28	−0.1 (5)	N9—N10—Ru1—N5	−133.0 (3)
C26—C27—C28—N9	−0.6 (4)	C26—N10—Ru1—N1	116.1 (13)
N12—C29—C30—C31	0.3 (4)	N9—N10—Ru1—N1	−57.1 (15)
C29—C30—C31—N11	−0.5 (4)	C29—N12—Ru1—N10	−142.1 (4)
C4—C5—N1—C1	−2.0 (6)	N11—N12—Ru1—N10	43.5 (2)
C4—C5—N1—Ru1	179.4 (3)	C29—N12—Ru1—N8	132.4 (4)
C2—C1—N1—C5	2.4 (6)	N11—N12—Ru1—N8	−42.0 (2)
C2—C1—N1—Ru1	−179.0 (3)	C29—N12—Ru1—N3	123.9 (12)
C2—C3—N2—C7	174.8 (4)	N11—N12—Ru1—N3	−50.5 (13)
C4—C3—N2—C7	−4.5 (6)	C29—N12—Ru1—N5	−55.1 (4)
C2—C3—N2—C6	0.3 (6)	N11—N12—Ru1—N5	130.4 (2)
C4—C3—N2—C6	−178.9 (4)	C29—N12—Ru1—N1	36.5 (4)
C11—C12—N3—C8	−1.6 (6)	N11—N12—Ru1—N1	−137.9 (3)
C11—C12—N3—Ru1	−174.3 (3)	C23—N8—Ru1—N10	128.6 (4)
C9—C8—N3—C12	1.5 (6)	N7—N8—Ru1—N10	−44.0 (2)
C9—C8—N3—Ru1	174.2 (3)	C23—N8—Ru1—N12	−144.6 (4)
C9—C10—N4—C14	176.7 (4)	N7—N8—Ru1—N12	42.8 (2)
C11—C10—N4—C14	−3.5 (6)	C23—N8—Ru1—N3	34.5 (4)
C9—C10—N4—C13	−1.6 (6)	N7—N8—Ru1—N3	−138.1 (3)
C11—C10—N4—C13	178.2 (4)	C23—N8—Ru1—N5	142.0 (8)
C18—C19—N5—C15	6.4 (5)	N7—N8—Ru1—N5	−30.6 (10)
C18—C19—N5—Ru1	−168.9 (3)	C23—N8—Ru1—N1	−56.5 (4)
C16—C15—N5—C19	−7.2 (5)	N7—N8—Ru1—N1	131.0 (2)
C16—C15—N5—Ru1	168.5 (3)	C12—N3—Ru1—N10	−26.3 (3)
C18—C17—N6—C21	−4.2 (6)	C8—N3—Ru1—N10	161.6 (3)
C16—C17—N6—C21	177.5 (4)	C12—N3—Ru1—N12	67.3 (13)
C18—C17—N6—C20	−171.7 (4)	C8—N3—Ru1—N12	−104.8 (12)
C16—C17—N6—C20	10.0 (6)	C12—N3—Ru1—N8	58.9 (3)
C24—C25—N7—N8	0.1 (4)	C8—N3—Ru1—N8	−113.2 (3)
C24—C25—N7—C22	173.9 (4)	C12—N3—Ru1—N5	−113.6 (3)
N11—C22—N7—C25	124.6 (4)	C8—N3—Ru1—N5	74.2 (3)
N9—C22—N7—C25	−112.3 (4)	C12—N3—Ru1—N1	154.6 (3)
N11—C22—N7—N8	−62.0 (4)	C8—N3—Ru1—N1	−17.6 (3)
N9—C22—N7—N8	61.1 (4)	C19—N5—Ru1—N10	−123.4 (3)
C24—C23—N8—N7	−0.1 (4)	C15—N5—Ru1—N10	61.5 (3)
C24—C23—N8—Ru1	−173.3 (3)	C19—N5—Ru1—N12	150.2 (3)
C25—N7—N8—C23	0.0 (4)	C15—N5—Ru1—N12	−24.9 (3)
C22—N7—N8—C23	−174.5 (3)	C19—N5—Ru1—N8	−136.8 (8)
C25—N7—N8—Ru1	174.9 (2)	C15—N5—Ru1—N8	48.1 (10)
C22—N7—N8—Ru1	0.4 (4)	C19—N5—Ru1—N3	−29.7 (3)
C27—C28—N9—N10	1.2 (4)	C15—N5—Ru1—N3	155.2 (3)
C27—C28—N9—C22	177.0 (3)	C19—N5—Ru1—N1	61.6 (3)
N11—C22—N9—C28	−113.2 (4)	C15—N5—Ru1—N1	−113.5 (3)
N7—C22—N9—C28	124.3 (4)	C5—N1—Ru1—N10	119.0 (13)
N11—C22—N9—N10	62.4 (4)	C1—N1—Ru1—N10	−59.5 (15)
N7—C22—N9—N10	−60.2 (4)	C5—N1—Ru1—N12	103.4 (3)
C27—C26—N10—N9	0.8 (4)	C1—N1—Ru1—N12	−75.1 (3)
C27—C26—N10—Ru1	−173.0 (3)	C5—N1—Ru1—N8	17.2 (3)
C28—N9—N10—C26	−1.2 (4)	C1—N1—Ru1—N8	−161.2 (3)
C22—N9—N10—C26	−177.6 (3)	C5—N1—Ru1—N3	−71.0 (3)
C28—N9—N10—Ru1	173.9 (2)	C1—N1—Ru1—N3	110.6 (3)
C22—N9—N10—Ru1	−2.4 (4)	C5—N1—Ru1—N5	−165.2 (3)
C30—C31—N11—N12	0.6 (4)	C1—N1—Ru1—N5	16.3 (3)
